# Introducing the suicide prevention trials database: A publicly available data repository of suicide prevention studies

**DOI:** 10.1111/sltb.13152

**Published:** 2025-02-03

**Authors:** Maya E. O'Neil, Stephanie Veazie, Danielle Krushnic, Sara Hannon, William Baker‐Robinson, Joren Adams, Kate Clauss, Joseph Constans, Jessica L. Hamblen, Vanessa C. Somohano, Lauren M. Denneson

**Affiliations:** ^1^ HSR Center to Improve Veteran Involvement in Care (CIVIC) VA Portland Health Care System Portland Oregon USA; ^2^ Department of Psychiatry Oregon Health & Science University Portland Oregon USA; ^3^ Department of Medical Informatics and Clinical Epidemiology Oregon Health & Science University Portland Oregon USA; ^4^ Northwest Mental Illness Research Education and Clinical Center VA Portland Healthcare System Portland Oregon USA; ^5^ Division of Epidemiology, School of Public Health University of California‐Berkeley Berkeley California USA; ^6^ School of Medicine Oregon Health & Science University Portland Oregon USA; ^7^ Office of Research and Development Veterans Health Administration Washington District of Columbia USA; ^8^ Department of Social, Behavioral, and Population Sciences School of Public Health and Tropical Medicine, Tulane University New Orleans Louisiana USA; ^9^ Department of Veterans Affairs National Center for Posttraumatic Stress Disorder White River Junction Vermont USA; ^10^ Department of Psychiatry Geisel School of Medicine at Dartmouth Hanover New Hampshire USA

**Keywords:** clinical trials, data repository, evidence synthesis, meta‐analysis, quantitative methods, suicide, suicide prevention

## Abstract

**Background:**

Healthcare, research, policy, and legislative stakeholders need timely, accurate, and detailed information on the effectiveness and potential harms of suicide prevention approaches. We created the Suicide Prevention Trials Database (SPTD) to provide a centralized, publicly accessible, detailed database of harmonized study‐level suicide prevention clinical trial data.

**Methods:**

We searched for randomized controlled trials (RCTs) of suicide prevention published from 1980 to 2023. Over 300 data variables were extracted from each RCT.

**Results:**

We identified a total of 140 unique RCTs in 180 articles. Most of the included RCTs compared two treatment arms (92%), and the remainder compared three arms (88%). Nearly half of the RCTs reported on Behavioral Interventions (49%), followed by Care Management, Follow‐up, or Monitoring (16%). Typically, the comparator condition was Treatment as Usual (53%). Interventions were most often delivered in person (61%) in an individual format (79%).

**Conclusions:**

The SPTD provides efficient, accurate, up‐to‐date access to a comprehensive suicide prevention trials database, which can be utilized by a range of stakeholders. It can reduce the time required for high‐quality systematic reviews and provides researchers, administrators, and funders with current data on the state of the literature.

## BACKGROUND

The suicide rate in the United States (U.S.) has been increasing since 1999, following a 15‐year decline (Curtin et al., 2016). During the past two decades (2000–2022), the suicide rate increased 36%, and over 49,000 people died by suicide in the U.S. in 2022 (Centers for Disease Control, 2024). This rise in suicides over the past several decades has been met with a proliferation of research on the treatment and prevention of suicidal ideation (i.e., thoughts of suicidal self‐directed violence) and suicidal behavior (i.e., suicidal self‐directed violence) (Crosby et al., 2015). Healthcare systems, including the Veterans Health Administration (VHA), have made suicide prevention a top priority for clinical care and research.

Healthcare, research, policy, and legislative stakeholders need timely, accurate, and detailed information on the effectiveness and potential harms of suicide prevention approaches. Given the vast number of prevention modalities (e.g., psychotherapy, psychopharmacology, complementary and integrative health approaches) and the tremendous diversity in participant and study characteristics, it is difficult to compile and synthesize findings in a timely manner. Efforts to systematically catalog and synthesize suicide prevention data have included systematic reviews (Nelson et al., 2017; O'Neil et al., 2012), clinical practice guidelines (Department of Veterans Affairs and Department of Defense, 2013, 2019), and clinical trial registries such as ClinicalTrials.gov. Even these high‐quality, systematic approaches can fall short if the evidence necessary to address a specific stakeholder question has not been recently searched, compiled, and harmonized. Additionally, it is common for clinical practice guidelines and systematic reviews to provide information about specific subsets of suicide prevention trials, but not about all trials, and not all in one place.

Data repositories are an efficient way to address some of the challenges of synthesizing research on complex, broad, high‐priority conditions like suicide. Successful databases have been established for closely related conditions. For example, a large database of study‐level data from randomized controlled trials (RCTs) of depression treatment has been maintained and updated annually for more than a decade (Cuijpers, 2017, 2019; Cuijpers et al., 2008). Researchers have used this database to conduct and publish more than 70 meta‐analyses, examining, for example, which psychotherapies are most effective, the efficacy of combined interventions, and the effects of specific therapies on targeted subgroups (for an overview, see Cuijpers, 2017).

In 2018, our research team developed the evidence tables for the “PTSD Trials Standardized Data Repository” (PTSD Repository) (O'Neil et al., 2020). This repository served as a model for the SPTD project. PTSD Repository evidence tables are available to the public for download as Microsoft Excel spreadsheets on both Agency for Healthcare Research and Quality (AHRQ) and National Center for PTSD websites, enabling access to these resources for purposes such as conducting systematic reviews, identifying research gaps and priorities, and serving as a resource for clinicians and patients to help identify what works for whom (O'Neil et al., 2023).

These successful repositories of depression and PTSD clinical trial data have advanced research by facilitating cross‐study comparisons and have encouraged researchers to incorporate standardized data elements in their studies. Further, these databases have addressed some of the gaps identified by policy stakeholders and have the potential to accelerate the work involved in systematic reviews, including living systematic reviews. No such database previously existed for trials of suicide prevention interventions, yet the need for timely and accurate information on the evidence to support suicide prevention approaches has never been greater. Therefore, through funding from the VA Clinical Sciences Research and Development Service, we developed the Suicide Prevention Trials Database (SPTD), which addresses the aforementioned gaps by providing a centralized, detailed, comprehensive database of harmonized, study‐level, publicly accessible suicide prevention trial data.

## AIMS

In the present manuscript, we describe the creation of the SPTD, https://www.hsrd.research.va.gov/centers/core/sprint/sptd.cfm, including development of a technical expert panel (TEP), our search strategy, and a summary of currently included studies. The abstracted data and methods documentation (including a detailed data dictionary and replicable search strategy) are publicly available and can be used for future systematic reviews or clinical practice guidelines, for identifying research gaps, and as an information resource for clinicians, policymakers, patients, and family members.

## METHOD

To establish the SPTD, we devised a two‐stage process in which the first stage (Phase 1) included RCTs, and the second stage (Phase 2) included all other relevant intervention study designs. In this paper, we report on Phase 1 of the SPTD.

### Agency for Healthcare Research & Quality Guidance

We followed applicable methods guidance from AHRQ to search for studies, screen for inclusion, and abstract data from included studies (AHRQ Methods Guide for Effectiveness and Comparative Effectiveness Reviews, 2014).

AHRQ guidance recommends the assembly of a technical expert panel (TEP) to guide study decisions. We convened a multidisciplinary TEP with 12 experts in a wide variety of suicide prevention interventions (e.g., pharmacologic, nonpharmacologic, complementary and integrative health interventions; community‐ or population‐level interventions; means safety approaches). TEP members were recruited based on significant contributions to the suicide prevention literature, clinical expertise, and leadership in the field such as involvement in clinical practice guideline development, conducting large‐scale clinical trials, or involvement in similar database development. TEP members represented the U.S. and Australia, and approximately two‐thirds held an affiliation with the U.S. Department of Veterans Affairs. The TEP meets approximately 2–3 times per year for each year of the project, and TEP members provide feedback on major project goals and deliverables (e.g., inclusion criteria, manuscript submissions, draft databases, data dictionaries, and other project documentation that will be maintained and disseminated over the course of this project). We followed guidance from the VA Evidence‐based Synthesis Program (ESP) and AHRQ Evidence‐based Practice Center (EPC) related to TEP involvement and feedback for this project. The project was submitted and approved by the Portland VA Institutional Review Board (IRB) as non‐human subjects research.

### Eligibility

Inclusion and exclusion criteria are described in Table 1. Phase 1 included data from RCTs with a primary aim of preventing suicide or suicidal self‐directed violence published from 1980 to 2023. Inclusion was limited to adult populations and studies published in English. Due to the large number (over 2000) of potentially relevant studies, we made decisions with the TEP about which types of studies to include at initial stages of database development and which to add at later stages. For example, we decided not to include cluster‐randomized studies in Phase 1. In a cluster randomized trial, providers, facilities (e.g., clinic, hospital), or communities are randomized into treatment and control groups, rather than randomizing individual participants (Lorenz et al., 2018). Cluster‐randomized studies are instead being included in the second phase of SPTD development.

**TABLE 1:** Phase 1 Inclusion and Exclusion Criteria.

	Inclusion criteria	Exclusion criteria
Population	Adults (≥18 years old) Will accept studies where average age of participants is ≥18	Children and adolescents (<18 years old)
Interventions	Interventions from studies whose primary aim is preventing suicidal ideation, suicide, or suicidal self‐directed violence	Interventions from studies whose primary aim is not preventing suicidal ideation, suicide or suicidal self‐directed violence
Comparators	No limitations applied (e.g., another intervention; usual care; no intervention)	None
Outcomes	Primary: Studies must report on suicide or suicidal self‐directed violence (e.g., fatal or non‐fatal suicide attempts) to be included Secondary: Suicide ideation and harms (e.g., any reported unintended consequences such as medication side effects) are additional outcomes of interest	Studies that do not report the primary outcome will be excluded
Timing	Any study duration and length of follow‐up	None
Settings	All	None
Study Design	Randomized controlled trials (RCTs)	Non‐randomized controlled studies, before‐after studies, narrative reviews, cross‐sectional studies, qualitative studies, editorials, and commentaries Selected systematic reviews will be considered as reference sources for studies to be reviewed for possible inclusion; however, data will be abstracted from individual studies, rather than from systematic reviews
Randomization Unit	RCTs in which individual participants are the unit of randomization will be included	Cluster‐RCTs in which the unit of randomization is the provider, clinic, community, or city will be excluded
Publication Language and Dates	English language articles 1980 to present	Non‐English language articles Unpublished data Publication date prior to 1980

For the *intervention* criterion, we excluded studies if authors did not either: (1) explicitly state suicide prevention was a primary aim of the study or (2) discuss the potential for the intervention to prevent suicide. Examples of excluded studies are those in which suicide was only discussed as a potential adverse event and studies in which the intervention was targeting another mental health condition and suicide was only mentioned as one of many measured outcomes and was clearly not the primary outcome of interest.

For the *outcome* criterion, studies had to report suicide deaths and/or nonfatal suicide attempt behavior outcomes. We included studies that reported “self‐harm” as suicidal behavior unless the studies clearly indicated that the behavior lacked suicidal intent (e.g., “non‐suicidal self‐harm,” “self‐harm without intent to die”). Our rationale is that definitions of self‐harm have changed over time, and therefore we wanted to include earlier studies that reported nonfatal suicidal behavior as “self‐harm.” We also excluded studies that reported only composite suicidal behavior and ideation outcomes (i.e., our focus was on including studies with suicidal behavior outcomes, not ideation or a composite where behavior could not be examined separately from ideation). Studies that reported on suicidal ideation outcomes *in addition to* separately reported suicidal behavior outcomes were included. We excluded studies that only reported suicidal ideation outcomes. While there is important information that can be gathered from trials targeting suicidal ideation, we recognized that ideation differs from behavioral outcomes. Excluding studies that only report suicidal ideation outcomes also enhanced the feasibility of developing the database with the available staff and resources. However, our team plans to expand the database in the future to include studies reporting suicidal ideation without suicide behavior outcomes.

Finally, studies had to report sufficient information on the suicide behavior outcome so that a between‐group effect size could be calculated. For example, a study that reported a dichotomous suicide behavior outcome like proportion of participants who experienced a suicide attempt would need to report either: (1) the between‐group comparison effect size or (2) the percentage of participants (or n/N of participants) in each group with the outcome to be included in the SPTD. Where a study reported “no adverse events”, we extracted 0 suicide deaths in each arm, giving sufficient data for effect size calculation.

### Search strategy

We searched reference lists of 12 relevant and comprehensive systematic reviews and the 2019 VA/DoD clinical practice guidelines on suicide prevention to identify potentially relevant RCTs published from 1980 to 2019. To identify RCTs published after these reviews, we searched MEDLINE, Cochrane Central Register of Controlled Trials, PsycINFO, and Cumulative Index of Nursing and Allied Health databases for RCTs published from 2020 to 2023. Our search strategy was developed by the leadership team and reviewed by a librarian with experience creating similar searches. The TEP reviewed and provided advice about search strategies.

### Study selection

Titles, abstracts, and full‐text articles were independently reviewed for eligibility by at least two reviewers. At the abstract level, any citation considered potentially eligible for inclusion by one reviewer was retrieved for full‐text review, and excluded abstracts were reviewed by at least one additional reviewer to ensure that no potentially relevant abstracts were missed. At the full text level, all articles were reviewed by at least two reviewers, and disagreements were resolved by consensus. A list of included and excluded studies was also reviewed by the TEP.

### Data abstraction

We constructed Microsoft Excel® tables with study characteristics and key study results for all included studies. Our final list of 353 abstracted elements was determined through consultation with the TEP as well as other stakeholders and operational partners from VA research and clinical operations including Clinical Science Research and Development (CSR&D), Health Systems Research (HSR), and the Office of Suicide Prevention (OSP). The data elements were abstracted from each RCT using single reviewer extraction (bachelor's or master's‐level research assistant/associate), with a second reviewer (master's‐level statistician) checking abstracted data for accuracy. A third reviewer (doctoral‐level subject matter or methods expert) provided a final check and addressed any reviewer questions.

Abstracted elements included multiple variables in the following categories: study design, setting, country, sample size, eligibility criteria, participant characteristics, intervention characteristics, eligible results, and sources of funding. Interventions were grouped into 21 categories. These included broad categories such as behavioral interventions, pharmacological interventions, and complementary and integrative health as well as categories that are more specific to suicide prevention research such as gatekeeper training, crisis hotlines and chatlines, and reducing access to lethal means. Results, including means, standard deviations, effect sizes, and other statistics, were abstracted from each study for included outcome variables. For more detail on the data elements abstracted, see Table 2.

**TABLE 2:** Abstracted data elements.

Data category	Elements extracted
Study identification	First author and year of publication; AHRQ‐style citation; PubMed, and ClinicalTrials.gov identifiers; funding source
Study characteristics	Country; study site type (e.g., VA/DoD); clinical/community setting; study design; subscale or symptom cluster data reporting; subgroup analysis reporting; psychotherapeutic intervention provider training; group therapy setting; psychotherapeutic and psychotropic co‐interventions
Suicide risk definition	Definitions of suicide risk used for study inclusion (e.g., ideation severity level, prior attempt status)
Population characteristics	Number of patients; % with suicide risk; baseline severity; suicide‐related symptom/diagnosis duration; % of patients in active duty military/veteran/community; % reintegrating Veterans; % homeless; mean age; gender; race; ethnicity; % treatment‐naïve; % with comorbid depression, SUD, and other associated comorbidities
Study and intervention description	Intervention classification according to socio‐ecological framework (individual, relationship, community, systems); number of patients randomized to each intervention; intervention name, description, dose/session length, frequency, and duration; intervention completion/adherence definition and % meeting criteria
Study interventions	Behavioral Interventions (e.g., Cognitive Behavioral Therapy, Dialectical Behavioral Therapy, and Problem‐solving psychotherapies); Pharmacological Interventions (e.g., Ketamine, Lithium, Clozapine, and selective serotonin reuptake inhibitor [SSR]) drugs); Nonpharmacological Biological (e.g., Electroconvulsive therapy [ECT], and neuromodulation); Care Management, Follow‐Up, or Monitoring (e.g., Collaborative Assessment and Management of Suicidality [CAMS], home visits, and collaborative care); Complementary and Integrative Health e.g., Mindfulness, Meditation, and Yoga); Technology‐Based Modalities (e.g., Mobile or web applications); Risk Assessment/Screening (e.g., Predictive analytics and suicidal ideation or risk screening strategies); Strengthening Access to Care/Increasing System Capacity (e.g., Reducing provider shortages in underserved areas and coverage of mental health conditions in health insurance policies); Reducing Access to Lethal Means (e.g., Firearm safety, bridge barriers, medication safety); Crisis Hotlines and Chatlines (e.g., Veterans Crisis Line), Gatekeeper Training (e.g., Army ACE, SAVE); Peer and Buddy Support Programs (e.g., Buddy intervention support group); Media Campaigns, Public Awareness, and Safe Reporting and Messaging about Suicide (e.g., Recommendations for reporting on suicide); Addressing Social Determinants of Health (e.g., Strengthen household financial security and housing stabilization); Postvention (e.g., StandBy support after suicide); Other (e.g., Social emotional learning programs and parenting skills and family relationship approaches); Placebo/Sham (e.g., Placebo like a sugar pill and sham procedure); Treatment as Usual (e.g., Treatment as Usual and Usual Care); Wait‐list or Other Passive Control (e.g., Wait‐list control and educational handout); Time and Attention Control (e.g., educational group sessions); Multiple Interventions (e.g., a combination of interventions listed above)
Suicide‐related outcomes: measures and analysis	Suicidal ideation severity outcome measure name; suicidal behavior type and method for assessment; method for handling missing data; statistical analysis type (e.g., ITT) and method; variables adjusted for primary outcome analysis
Suicide‐related outcomes: primary outcome by group	Number of patients assessed; mean measure score; measure change; within‐group effect size; risk‐related change, and clinically meaningful response
Suicide‐related outcomes: across‐group comparison	Effect sizes for primary outcome measurement, risk‐related change, and clinically meaningful response
Suicide‐related outcomes: between‐group comparison	Score difference and effect sizes for primary measure, risk‐related change, and clinically meaningful response
Suicide‐related outcomes: secondary outcomes	Names of other suicide‐related outcomes; baseline score; effect size for between‐group score difference
Other outcomes and harms	Measurement used to assess Depression, Anxiety, Substance Use, Sleep, Anger, Quality of Life, and Functioning; effect size for between‐group score difference; % (n/N) of SAE, WAE

We also created a data dictionary with operational definitions for each data element to guide data abstraction, ensure cross‐study variable standardization and harmonization, and facilitate dissemination and use of the database by stakeholders. TEP members reviewed and provided advice on variables for abstraction and data dictionary definitions at multiple times during the development process.

#### Clarification on race, ethnicity, sex and gender, and suicide attempt history

It is important to note that all data abstraction was limited to what was defined and reported in the included studies, and therefore when data did not fit within the pre‐specified grouping categories in the SPTD, there were gaps in abstracted data. This was particularly true for some demographic variables given how varied certain definitions were (e.g., race/ethnicity) across studies. Users of the database should keep in mind that these data gaps likely represent different ways of reporting data, and not necessarily that these data weren't collected in the studies.

Race and ethnicity are social constructs that vary by region and country. Due to this wide variation and the need to create comparable data across studies, the *race and ethnicity* variables were abstracted according to United States Office of Management and Budget (OMB) categories. For race, OMB has 5 categories: Asian, American Indian Alaska Native, Black, Native Hawaiian or Other Pacific Islander, White. In addition to these categories, SPTD also has a field for “Other.” The OMB category for ethnicity is a single category of “Hispanic or Latino.” We included two qualitative columns for race and ethnicity to capture these data when the reported data did not fit into OMB categories. This was especially helpful in the case of international studies reporting race and ethnicity and to highlight studies that combined race and ethnicity into one category.

The *percent of women* variable is defined as the percent of study participants who were categorized as female (sex at birth) and/or women. In almost all cases, not enough information is provided in the studies to discern gender identity. Qualitative columns for gender were also included to allow data in more inclusive and diverse gender identity categories to be extracted if reported by the study.

The *percent of sample with suicide attempt history* variable indicates the percent of participants in the study sample with one or more suicide attempts or self‐harm events prior to study enrollment.

#### Risk of bias

Risk of Bias (RoB) is the likelihood that choices in the design, method, analysis, or reporting of a study have introduced systematic error or “bias” in the reported intervention effect (Higgins et al., 2011). We used Cochrane's RoB 2 tool to evaluate RoB in reported results for suicide‐related outcomes in each study. RoB was independently assessed by two team members across five domains: bias due to randomization, bias due to deviations from intended interventions, bias due to missing outcome data, bias in the measurement of the outcome, and bias in the selection of reported results (Sterne et al., 2019). Each domain was rated as *Low*, *Some Concerns*, or *High* RoB. The two team members then discussed the domain ratings to come to consensus, which were then combined into an overall RoB rating for each result assessed. Overall RoB assessments were assigned a *Low* rating if all domains were rated as *Low*, a *Some Concerns* rating if at least one, but no more than two domains were rated as *Some Concerns* and no domains were rated *High*, and a *High* rating if three or more domains were rated as *Some Concerns* or any domain was rated as *High*. For each study included in SPTD, reported results were assessed for up to two outcomes, with priority given to assessing suicide behavior outcomes. If multiple results were reported for an outcome (e.g., results reported at multiple study timepoints), the reported result with the lowest overall RoB rating was included in the final database. Importantly, RoB was evaluated separately for each reported result in a study; thus, studies which report multiple suicide‐related outcomes may have different RoB ratings depending on which outcome measure or result is being considered.

## RESULTS

### Search

**FIGURE 1:**
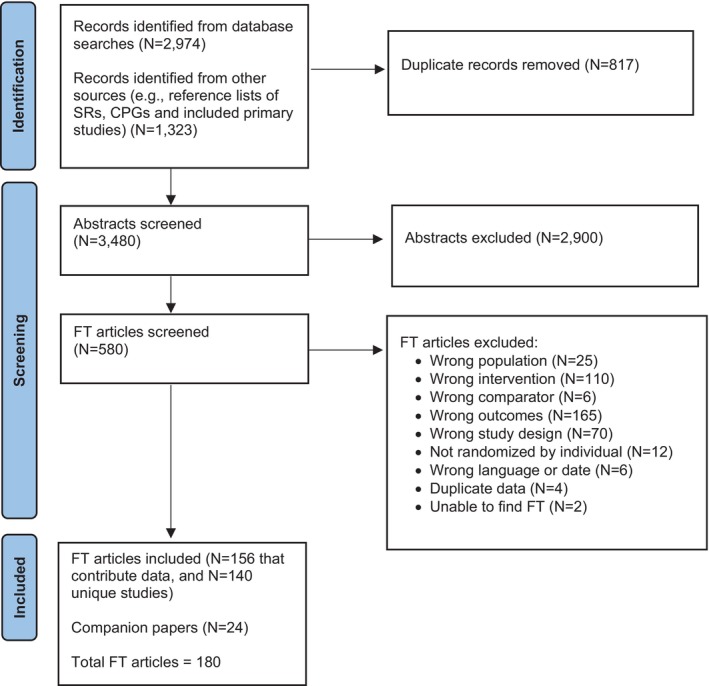
Literature flow diagram.

Our RCT search of primary studies published from 2020 to 2023 yielded 2974 records with 817 identified as duplicates and removed. An additional 1323 studies were identified through reviews of reference lists of systematic reviews, clinical practice guidelines, and primary studies published from 1980 to 2019, resulting in 3480 abstracts being screened after duplicates were removed. During abstract screening, 2900 records were excluded, leaving 580 records for full‐text review. At the full‐text level, 400 records were excluded, leaving 180 articles for 140 unique RCTs included in Phase 1 of the SPTD; 156 articles contribute data and 24 are companion publications without usable data. Search results and exclusion criteria are presented in Figure 1.

### What suicide prevention interventions have been studied?

#### Primary interventions

Most of the suicide prevention trials had two treatment arms (92%), although 11 studies (88%) included three arms. In the 140 RCTs included in Phase 1 of the SPTD, interventions from ten primary intervention categories were studied. Behavioral Interventions (e.g., cognitive behavioral therapy, dialectical behavior therapy, and problem‐solving psychotherapies) were the most common (*n* = 68, 49%), followed by Care Management, Follow‐Up, or Monitoring (*n* = 23, 16%, e.g., collaborative assessment and management of suicidality (CAMS), home visits, and collaborative care). Additionally, 16 studies (11%) focused on Pharmacological Intervention (e.g., ketamine, lithium, clozapine, and selective serotonin reuptake inhibitors). There are 21 studies in the SPTD with a primary intervention category of Multiple; almost all of these (*n* = 20; 95%) included behavioral intervention components. The most common interventions to be combined were Behavioral Interventions and Care Management, Follow‐Up, or Monitoring (*n* = 16, 76%).

### Intervention characteristics

Most studies tested a primary intervention in an individual format (*n* = 110, 79%), followed by group combined with individual (*n* = 17, 12%), and group‐alone (*n* = 6, 4%). The most common delivery methods were in‐person (*n* = 86, 61%), followed by in‐person and phone (“In‐person; Phone”; *n* = 22, 16%), and technology‐alone (*n* = 7, 5%). Most studies used Treatment as Usual as the control/comparison group type (*n* = 74, 53%).

### Interventions over time

Over the years, there have been increasing numbers of RCTs published on suicide prevention interventions, and there have also been interventions tested from more categories of suicide prevention. Between 1980 and 1989 there were five studies published that met criteria for inclusion in the SPTD. One decade later, from 2000 to 2009, a total of 25 studies were published including the first study of a complementary and integrative health intervention. Between 2010 and 2019, a total of 66 studies were published in which treatments from eight primary intervention categories were tested. The new primary intervention categories studied during this decade included nonpharmacological biological, technology‐based modalities, peer and buddy support programs, and addressing social determinants of health. Finally, in the most recent three years, from 2020 to 2023, there were 33 studies published examining treatments from seven primary intervention categories.

### Who has been studied?

#### Demographics

This first phase of SPTD development only included studies on adults. The database includes aggregated data from a total of 68,499 participants across the 140 included studies. The average of study‐reported mean ages was 34.54 years, derived from 120 studies (86% of included studies). In phase 1, 24 studies (17%) reported data on race but not ethnicity, 33 studies (24%) reported data on race and ethnicity, and 83 studies (59%) did not report race/ethnicity data in a way that could be categorized into OMB categories. Of these 83 studies, only 4 reported on race or ethnicity in such a way that we could not extract the information according to the SPTD categories; the remaining 79 did not report any race or ethnicity data. Most studies (82; 59%) had a sample including between 50% and 79% women. Nine studies enrolled 100% women, and only two studies enrolled 100% men. Studies took place in 26 countries; the three most common countries were the United States (*n* = 47, 34%), the United Kingdom (*n* = 17, 12%), and Australia (*n* = 11, 88%).

#### Mental health and suicide‐related variables

**FIGURE 2:**
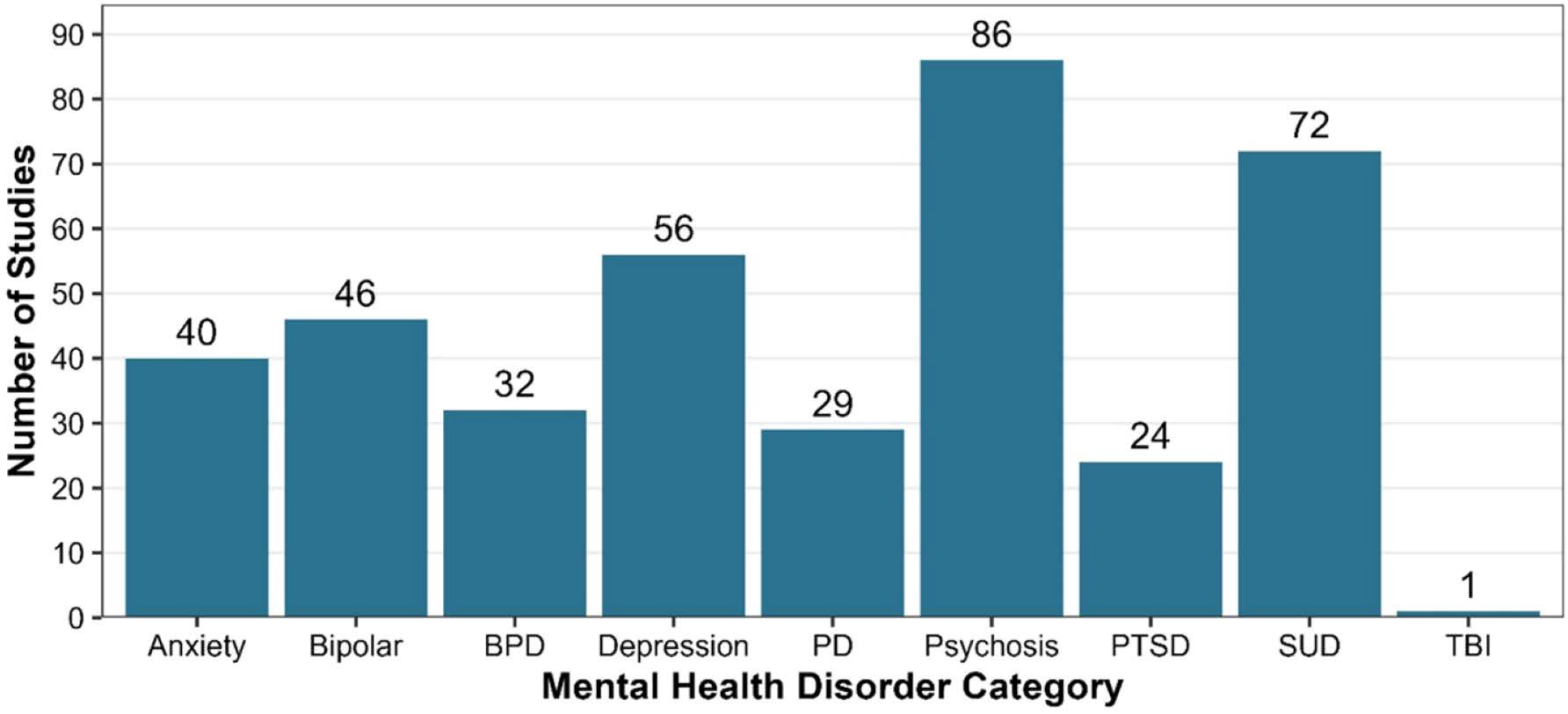
Studies reporting mental health disorders of enrolled participants. Phase 1 Dataset, *N* =140 studies (studies could include more than one mental health disorder category). BPD, borderline personality disorder; PD, personality disorder; PTSD, posttraumatic stress disorder; SUD, substance use disorder; TBI, traumatic brain injury.

The percent of participants with a suicide attempt history was reported in 104 (74%) studies. There were 61 (44%) studies where 100% of the study sample had a history of a suicide attempt or self‐harm event at baseline. All studies in phase 1 of the SPTD had a suicide prevention stage of either indicated (i.e., targeted participants with a history of suicidal ideation, attempts, or psychiatric hospitalization) or selected (i.e., targeted participants with a history of mental health disorders or other characteristics associated with a higher risk for suicide, but no history of suicidal ideation, attempts, or psychiatric hospitalization). Indicated was the most common suicide prevention stage (*n* = 118, 84%). The SPTD also tracks reporting of the following baseline mental health‐related conditions: anxiety, bipolar disorder, borderline personality disorder, other personality disorders, psychosis, posttraumatic stress disorder, substance use disorders, and traumatic brain injury (see Figure 2 for details related to these conditions).

#### Risk of bias

As described, we evaluated RoB for up to two suicide‐related outcomes reported per study, resulting in 252 RoB ratings for 140 RCTs. Regarding overall RoB, 163 (65%) were rated *High*, 49 (19%) were rated *Some Concerns*, and 40 (16%) were rated *Low*. For studies using suicide deaths as an outcome (*n* = 85), 50 (59%) were rated *High*, 18 (21%) were rated *Some Concerns*, and 17 (20%) were rated *Low*. For studies using suicide attempts as the outcome (*n* = 99), 63 (64%) were rated *High*, 22 (22%) were rated *Some Concerns*, and 14 (14%) were rated *Low*. Finally, for studies using suicide ideation as the outcome (*n* = 43), 34 (79%) were rated *High*, 7 (16%) were rated *Some Concerns*, and 2 (5%) were rated *Low*.

## DISCUSSION

The SPTD is a potentially powerful resource for suicide prevention research. This publicly available database (available at https://www.hsrd.research.va.gov/centers/core/sprint/sptd.cfm) provides efficient, accurate, and up‐to‐date access to a comprehensive database of suicide prevention trials in adults which can be used for a variety of purposes by a broad range of stakeholders. First, the SPTD is designed to allow researchers to conduct systematic reviews and meta‐analyses of important suicide prevention topics much faster than conducting searches, culling the extensive literature base, and abstracting data each time such a review is needed. The transparent (e.g., public, detailed data dictionary) and gold standard methods (e.g., following AHRQ and VA ESP methods guidance, using Cochrane ROB 2 assessment) were chosen to allow researchers to understand exactly what to expect when using the database for systematic review purposes. The detailed data extraction of over 300 variables, as well as the standardized definition of variables, allows researchers to use the database to conduct high‐quality systematic reviews on different subtopics relevant to suicide prevention. For example, the SPTD can be used to examine interventions tested primarily in women or compare the efficacy of different types of interventions such as DBT and problem‐solving therapy. Administrators and funders can quickly identify what interventions have been studied in which populations, helping them to select interventions with the strongest evidence for implementation or identify interventions or subpopulations in need of further support for additional research.

While the database itself is primarily designed as a tool for researchers and other policy‐related data teams, affiliated products are also available to ensure relevance for a broader clinical and patient/caregiver audience. Our team has developed “Data Stories” (available at https://www.hsrd.research.va.gov/centers/core/sprint/SPTD‐Data‐Story1‐What‐Interventions‐Have‐Been‐Studied.pdf and https://www.hsrd.research.va.gov/centers/core/sprint/SPTD‐Data‐Story2‐Who‐Has‐Been‐Studied.pdf) as a way to share key data summaries and takeaways from the SPTD. We have also developed online, interactive data visualizations so that stakeholder groups like patients, caregivers, clinicians, trainees, or others in need of up‐to‐date information on what works for whom can access these summary data without extensive statistical knowledge or software (https://www.hsrd.research.va.gov/centers/core/sprint/intervention‐dashboard.cfm).

Finally, the SPTD provides a myriad of data summaries on what is contained within the body of available suicide prevention trial literature. Notably, most studies have been individually administered behavioral interventions, though recent trends include a growing focus on other intervention types including Nonpharmacological Biological, Technology‐Based Modalities, Peer and Buddy Support Programs, and Addressing Social Determinants of Health interventions. The database also provides information on who has been studied, tracking demographic characteristics of participants such as race/ethnicity, gender, and military/civilian status as well as suicide attempt history and mental health diagnoses.

The SPTD will be updated as new studies continue to be published. In these planned updates, an updated literature search and abstract review, full text review, and data abstraction will be completed for all newly added articles. At each update, accompanying documents such as the data summaries, interactive visualizations, and future publications will be date‐stamped so that users can track updates.

### Limitations

While the SPTD has the potential to significantly speed up the process of conducting systematic reviews, there remain significant limitations to this resource. First and foremost, the SPTD only contains *study*‐*level* data, not individual participant‐level data. This means that only aggregate data are included, and we are not able to parse out findings in a more granular manner. For example, while studies may include people of various genders or racial/ethnic identifications, if the primary study does not present separate results for each of those subgroups, there is no way to include subgroup‐specific results in the SPTD, and instead, only aggregate data and results for the overall sample can be included in the database.

Additional limitations are primarily related to the inclusion/exclusion criteria for the database. Due to the large number of studies on suicide prevention, the scope of work for Phase 1 focused on individually randomized RCTs conducted in adult participants. Additionally, we focused on studies including a *behavioral* suicide‐related outcome (i.e., suicidal self‐directed violence such as death by suicide or suicide attempt); studies reporting on only suicidal ideation were excluded. The SPTD also includes only interventions designed to prevent suicide and does not include studies of interventions focused on treating related conditions (e.g., depression, bipolar disorder, borderline personality disorder) even if suicide behavior outcome data or adverse events are reported. Though these data are relevant to preventing suicide, our focus was on trials of interventions designed to prevent suicide/suicidal self‐directed violence in adults.

### Future directions

There are many possible ways that this database could be expanded in the future. Already underway is the Phase 2 expansion of the database to include nonrandomized trials of suicide prevention interventions. Because of the potential importance of community‐level, large‐scale interventions to prevent suicide (e.g., bridge barriers, screening implementation across large healthcare systems) that are difficult or impossible to study using an RCT study design, the TEP recommended including these and other nonrandomized studies in the second phase of SPTD development. Inclusion criteria and the types of data extracted for these studies were developed with the TEP and account for differences in nonrandomized studies' design, implementation, and potential biases.

Another recently initiated expansion of this project is the inclusion of individual participant‐level data. While compiled and harmonized study‐level data provide a powerful tool for researchers, policymakers, providers, and patients, there are also significant limitations of relying on study‐level, aggregate data. Leveraging individual participant‐level data makes the most robust use of existing data from suicide prevention intervention RCTs. Specifically, individual participant‐level data facilitates answering key questions about what works for whom, advancing research findings for historically underrepresented and marginalized groups by combining small samples across trials to elucidate findings that are otherwise suppressed in aggregate results. Our team's work expanding the SPTD to include individual participant‐level data was initiated in 2024. As this expansion project advances over the next 5 years, updates and interim results will be made publicly available on the SPTD website.

## CONCLUSION

The SPTD addresses the need for a comprehensive, accurate, up‐to‐date repository of suicide prevention RCTs. Not only does it allow researchers to conduct timely systematic reviews, but it also gives administrators and policymakers access to current, comprehensive information on suicide prevention trials. Further, additional products are being developed to make the SPTD accessible to clients and care providers, which will help enhance health literacy related to suicide. Finally, work is already underway to expand the utility of this new resource for suicide prevention research, policy, and clinical care.

## FUNDING INFORMATION

Primary Funding Source is Clinical Science Research & Development (Award # SDR‐SPTD‐20S). We thank the Technical Expert Panel for their assistance with developing the database. All members are listed on the Suicide Prevention Trials Database website, https://www.hsrd.research.va.gov/centers/core/sprint/sptd.cfm.

## CONFLICT OF INTEREST STATEMENT

There are no conflicts of interest to declare.

## DATA AVAILIBILITY STATEMENT

The data that support the findings of this study are openly available at https://www.hsrd.research.va.gov/centers/core/sprint/sptd.cfm.
